# Antimicrobial Activity of Honey and Propolis from Alba County, Romania

**DOI:** 10.3390/antibiotics13100952

**Published:** 2024-10-10

**Authors:** Mihaela Laura Vică, Mirel Glevitzky, Gabriela-Alina Dumitrel, Maria Popa, Ioana Glevitzky, Cosmin Adrian Teodoru

**Affiliations:** 1 Department of Cellular and Molecular Biology, “Iuliu Hațieganu” University of Medicine and Pharmacy, 400012 Cluj-Napoca, Romania; 2 Faculty of Exact Science and Engineering, “1 Decembrie 1918” University of Alba Iulia, 510009 Alba Iulia, Romania; 3 Sanitary Veterinary and Food Safety Directorate of Alba County, 510217 Alba Iulia, Romania; 4 Faculty of Industrial Chemistry and Environmental Engineering, Politehnica University of Timisoara, 300223 Timișoara, Romania; 5 Clinical Surgical Department, Faculty of Medicine, “Lucian Blaga” University of Sibiu, 550025 Sibiu, Romania

**Keywords:** honey bees, propolis, chemical analysis, antibacterial and antifungal activity, statistics

## Abstract

Investigating the quality of bee products obtained across different geographical regions and analyzing their antimicrobial activity is of significant interest to various scientific disciplines. This study focuses on comparing the antimicrobial activity of honey and propolis samples from different areas of Alba County, Romania. The quality parameters of five samples of two types of bee products (honey and propolis) were assessed. Then, the samples were tested to comparatively determine their antimicrobial properties against 12 species of bacteria (*Escherichia coli*, *Salmonella typhimurium*, *Salmonella enteritidis*, *Salmonella anatum*, *Salmonella choleraesuis*, *Pseudomonas aeruginosa*, *Pseudomonas fluorescens*, *Staphylococcus aureus*, *Staphylococcus epidermidis*, *Bacillus cereus*, *Bacillus subtilis*, and *Listeria monocytogenes*) and 7 fungal strains (*Candida albicans*, *Aspergillus niger*, *Aspergillus flavus*, *Penicillium chrysogenum*, *Rhizopus stolonifer*, *Fusarium oxysporum*, and *Alternaria alternata*). Of the bacterial strains, the most sensitive to the action of honey samples were the two strains of *Staphylococcus* followed by *P. fluorescens*. The two strains of *Pseudomonas* and *L. monocytogenes* were the most sensitive to the activity of propolis. Of the fungal strains, *F. oxysporum* was the most sensitive to the actions of both honey and propolis, followed by *P. chrysogenum* in the case of honey samples and the two *Aspergillus* strains in the case of propolis. These findings indicate that bee products are rich sources of bioactive compounds exhibiting strong antimicrobial properties and significant potential for the development of new phytopharmaceutical products.

## 1. Introduction

Bee products contribute to increased energy and an enhanced immune system [1] and have numerous therapeutic effects. Among these, antibacterial [2,3], antifungal [4], antiviral [5], hepatoprotective [6], anti-inflammatory [7], anticancer [8], immunostimulant [9], analgesic [10], and cicatrizing [11] activities have been reported. The concept of apitherapy centers on utilizing bee products as a potent adjuvant to support the management and healing of a wide range of medical conditions within alternative medicine practices [12].

Honey is a natural food produced by bees through the enzymatic transformation of floral nectar or extrafloral juices [13]. It is a mixture rich in simple sugars (fructose and glucose) that offers many nutritional benefits and contains many minerals, vitamins, antioxidants, amino acids, and enzymes (such as invertase and amylase) as well as hydrogen peroxide, antioxidants, lysozyme, polyphenols, phenolic acids, flavonoids, methylglyoxal, and bee-derived peptides [14,15,16,17,18,19]. Honey has been used for therapeutic purposes since ancient times. It is even depicted in Stone Age paintings from about 10,000 years ago [20]. Ancient civilizations, including the Egyptians, Assyrians, Chinese, Greeks, and Romans, used honey in various skin and wound treatments as well as for gastrointestinal aliments [21,22]. Later, honey was used in a mixture with vinegar to relieve pain or with water to satisfy thirst; it was also used in a mixture with different medicinal substances to reduce fever [23]. Currently, honey is also used in modern medicine and the cosmetic industry.

Propolis is the resinous material that bees use to seal their nests, and it is used in both traditional and homeopathic medicine due to its many benefits in fighting infections [24]. The use of propolis as a medicinal product originated in ancient Greece, Egypt, and Assyria [25]. The first medicinal uses of propolis date back to ancient Greece, around 350 BC, when it was used to treat dental abscesses. The Egyptians used it for mummification, and the Assyrians used it to heal skin wounds [26]. It contains more than 300 compounds (the most important being polyphenols, sesquiterpene quinines, coumarins, essential oils, amino acids, and inorganic compounds) and is a mixture of substances with biological activity, wax, and secretions of honey bees [27]. Propolis has beneficial effects on the body given its antiviral, antibacterial, antiseptic, antifungal, antiparasitic, anti-inflammatory, antioxidant, cicatrizing, and slightly anesthetic activities. It also demonstrates strong immunomodulatory activities [28]. Some of the substances present in propolis can even be useful in the treatment of cancer [29].

Romanian bee products, especially honey, have a remarkable reputation throughout the world due to their superior quality [30]. Romania is one of the main honey producing countries in the EU [31]. It is known for its diverse flora, with vast grasslands, centuries-old forests, and hills covered in wildflowers. This variety of plants and flowers specific to the area contribute to the distinct aroma and taste of bee honey. Thus, Romanian honey is rich in nutrients and substances beneficial for health [32,33]. In addition, the favorable climatic conditions in Romania are another important factor for obtaining the superior quality of bee honey [34]. Sunny and mild summers, as well as cold and long winters, ensure a proper flowering and pollination cycle [35]. Thus, the bees have access to constant sources of nectar and pollen, contributing to the abundant production of honey [36].

Beekeeping in Romania faces many challenges due to the impact of climate change and the use of pesticides in agriculture. However, in the last decade, Romania has reached the rankings of the main producers of honey in the European Union. Along with Spain, Germany, and Hungary, Romania is one of the EU’s most important honey producers [37].

Table 1 displays the amount of honey that is produced both nationally and in Alba County.

Due to climate change, Romania’s honey output decreased in 2022 and 2023. The cold, rainy spring severely damaged 60% of the acacia trees, and the subsequent drought caused the blossoms to wither and destroyed more than 50% of the sunflower plants. Furthermore, beekeeping productivity was severely impacted by catastrophic weather events in 2024, with a loss of 40–45% of the nation’s honey production [40].

Regarding propolis, the hive’s needs at the time define the quantity generated by bees. Using properly controlled techniques, beekeepers can boost the hives to produce up to 0.5 kg of propolis in a beekeeping season [41]. Both the Codex Alimentarius Commission [42] and the European Union through Directive 110/2001 [43] have established several quality standards that have been recognized and listed as “Current International Standards for Honey”.

Although the antimicrobial properties of honey are well-known, the exact mechanisms of action remain incompletely understood. It is well-established that concentrated sugar solutions can inhibit bacterial growth due to their high osmolality. However, natural honey exhibits a stronger inhibitory effect against clinically significant Gram-positive cocci, such as methicillin-resistant *Staphylococcus aureus* (MRSA) and certain enterococci, compared to artificial honey solutions. This suggests that bacterial inhibition by honey is not solely dependent on osmolality [44]. Honey’s broad-spectrum antimicrobial properties, which include antibacterial, antifungal, antiviral, and antimycobacterial effects, can be attributed to several factors, including honey’s low pH (acidity), osmotic effect, high sugar content, and the presence of bacteriostatic and bactericidal compounds. Additionally, honey’s ability to enhance cytokine release, along with its immune-modulating and anti-inflammatory properties, contributes to its antimicrobial action, which operates through multiple mechanisms [45].

The analysis of the antimicrobial mechanisms of propolis suggests that it affects the permeability of microbial cell membranes, disrupts membrane potential and adenosine triphosphate (ATP) production, and reduces bacterial mobility [46]. The antimicrobial effect of propolis is due to its chemical composition. Artepillin C (3,5-diprenyl-p-coumaric acid) is a phenolic compound found in propolis, specifically a prenyl derivative of p-coumaric acid. Research conducted in Brazil by Veiga et al. revealed that ethanolic extracts of propolis contain a higher concentration of artepillin C. These ethanolic extracts also demonstrated significant antibacterial activity against MRSA [47]. Flavonoids in propolis, including quercetin and rutin, are key components responsible for its antimicrobial and antiviral activity. These flavonoids have been identified in both ethanolic and aqueous extracts of propolis, which helps explain the properties of propolis [48].

In previous studies, we focused on bee products from different areas of Romania. This research reveals the properties of samples from different regions of Alba County, Romania. The present paper focuses on the determination of the main physico-chemical parameters of 5 bee honey and 5 propolis samples. The purpose of this study was to test the antimicrobial effect of these bee products on 12 bacterial and 7 fungal strains in order to identify the strains for which the analyzed honey or propolis demonstrates the best efficiency for use as an alternative therapy. In addition, statistical analysis was conducted to highlight whether the diameter of the inhibition zones is influenced by the geographical origin of bee products and by the strain used. At the same time, the correlation between the total phenolic and flavonoid content and the antimicrobial activity of bee products was highlighted.

## 2. Results

The values of the physico-chemical parameters of the polyfloral honey (H) samples collected from Alba County are presented in Table 2.

The water content of the honey samples lies within the maximum limit of 20%. The a_w_ of the polyfloral honey analyzed is lower than 0.6. According to Romanian STAS 784/3-2009 [49], it is recommended that the ash content not exceed 0.5%. The pH values, which are crucial during honey extraction due to its impact on texture and stability, range from 3.85 to 4.14. The pH value of honey together with its free acidity prevent the growth of various specific microorganisms. The values of these parameters are high only when the processing or storage of the honey is inadequate. The 5-hydroxymethylfurfural (HMF) content in the honey samples is below the recommended threshold of 1.5 mg/100 g and within the acceptable quality limit of 4.0 mg/100 g for honey that has been liquefied and packaged in jars. The total phenolic content of honey samples ranged from 63.51 to 98.49 mg GAE/100 g honey, while the total flavonoid content was 2.57 to 5.35 mg QE/100 g honey.

For the polyfloral honey samples analyzed, the values of the physico-chemical parameters are within the reference ranges presented in the EU Directive [43] and Romanian national regulations [50].

The values of the physico-chemical parameters of the propolis (P) samples collected from Alba County are presented in Table 3.

The moisture content in propolis ranges between 4.38 and 6.31%. The water activity of the propolis samples ranges from 0.62 to 0.71. The total ash content of propolis indicates that the extract obtained from the maceration process contains minerals. The phenolic content of the propolis samples from Alba County, Romania, varied from 149.2 to 180.4 mg GAE/g, while the flavonoid content ranged from 68.33 to 80.72 mg QE/g.

Table 4 shows the diameters of the inhibition zones for the bacterial strains obtained with polyfloral honey samples from Alba County.

As can be seen in Table 4, the most sensitive strains to the antibacterial action of honey were the two staphylococci, followed by *P. fluorescens*, *B. subtilis*, and *P. aeruginosa*. Some of the honey samples analyzed (mainly the I H sample) did not have an antibacterial effect on all strains. Some of the strains of *Salmonella* spp. were resistant to the action of some honey samples. Samples II H and III H, from the sub-mountain and mountain areas, were the most effective regarding the antibacterial activity.

Table 5 shows the diameters of the inhibition zones on the bacterial strains for propolis samples from Alba County.

Regarding the antibacterial activity of propolis extracts, it can be observed that all propolis samples had an inhibitory effect on all the strains tested. In some cases, the diameters of the inhibition zones were even larger than those produced by the antibiotic. The most sensitive strains to the action of propolis were the two strains of *Pseudomonas*, *L. monocytogenes*, and the strains of *Staphylococci* and *Bacillus* spp. For the strains of *Pseudomonas* spp. and *L. monocytogenes*, all samples yielded diameters of the inhibition zones that were larger than the antibiotic control. The propolis samples II P and III P, as well as the honey samples II H and III H, were collected from the same regions and demonstrated the highest antibacterial properties.

Table 6 shows the diameters of the inhibition zones of the fungal strains obtained with polyfloral honey samples from Alba County.

With two exceptions (sample I H in the case of *C. albicans* and sample V H in the case of *A. alternata*), all honey samples had an antifungal effect on the studied strains. The strongest effect was observed on the strain of *F. oxysporum*, followed by *P. chrysogenum* and *A. flavus*. As in the case of bacterial strains, samples II H and III H had the strongest antifungal effect.

Table 7 shows the diameters of the inhibition zones for the fungal strains obtained with propolis samples collected from Alba County.

As can be seen from Table 7, all propolis samples inhibited the proliferation of fungi. In some cases (such as *R. stolonifer* and *A. alternata*), the diameter of the inhibition zones were greater than that produced by the antifungal substance as a control. As noted for honey, the most sensitive strain was *F. oxysporum*, but it was followed by the two strains of *Aspergillus* spp. and *P. chrysogenum*. As in the case of honey, samples II P and III P showed the strongest inhibitory effect on the studied strains.

The results for the minimum inhibitory concentration (MIC) and minimum bactericidal concentration (MBC)/minimum fungicidal concentration (MFC) for the honey samples with inhibitory effects are shown in Supplementary Tables S1 and S2. The antimicrobial activity was observed at dilutions of up to 1/16 (*w*/*v*) for some bacterial strains. Some honey samples demonstrated antifungal activity even at a dilution of 1/32 (*w*/*v*); however, this activity was not observed for all strains.

The results for MIC and MBC/MFC in the cases of propolis extracts are shown in Supplementary Tables S3 and S4. With the exception of samples I P and IV P, most exhibited antimicrobial activity at concentrations up to 6.25 mg/mL; however, this was not noted for all strains. Several samples showed inhibitory effects at concentrations of 3.12 mg/mL for certain strains. Notably, samples II P, III P, and V P had an inhibitory effect at concentrations as low as 1.56 mg/mL. Specifically, sample II P was effective against strains of *P. aeruginosa* and *P. fluorescens*; sample III P inhibited *P. aeruginosa*, *B. subtilis*, *L. monocytogenes*, and *F. oxysporum*; and sample V P inhibited *P. fluorescens* and *B. cereus*.

## 3. Discussion

The composition and properties of honey and propolis are such that these products inhibit the growth of microorganisms. Honey’s high sugar content, low water activity, high osmotic pressure, low pH, and hydrogen peroxide production create an unfavorable environment for the survival or growth of microflora [51].

In accordance with the 784/3-2009 National Standard, the primary physico-chemical requirements, along with the recommended limits for polyfloral honey sold on the Romanian market, are presented in Table 8.

Comparatively, the values provided by the European legislation are also presented [38].

European and international norms are more tolerant regarding the physico-chemical quality conditions of polyfloral honey. The honey from Alba County, Romania, has a chemical composition and particular physico-chemical properties like the honey from Transylvania described by Iancu et al., 2012 [53]. The values obtained are similar to those reported in the literature [54].

The A_w_ value is below 0.6, a value at which microbial growth is inhibited and fermentation due to the prevention of osmophilic yeasts. The ash content gives information about the mineral content, which is determined by the botanical origin of the plants [55]. The average ash content in this study was 0.34% and lies within the range of 0.1% to 0.5% for European polyfloral honey [56,57].

5-Hydroxymethylfurfural is naturally formed in honey during the heating process or long-term storage [58]. The values obtained (0.47–1.39 mg/100g) belong to the category of fresh honey, not thermally treated/liquefied honey.

Polyfloral honeys can have variable phenolic content in Europe, but the values are typically between 30 and 120 mg GAE/100 g [59,60,61]. The values obtained for the polyfloral honey samples from Romania evaluated in this study are within these levels, exhibiting a range of 63.51 to 98.49 mg GAE/100 g.

The flavonoid content of polyfloral honey from Europe is between 5 and 50 mg quercetin equivalent (QE)/100 g honey [62,63]. The results obtained (2.57–5.35 mg QE/100 g) are slightly below the European average. Sample III from Abrud area had the highest content of flavonoids at 5.35 mg QE/100 g among the samples analyzed.

Several studies have shown that the moisture content in propolis varies between 0.96% and 2.97%. The samples from Romania are similar to those reported from different districts in Bulgaria, which showed a moisture content between 0.98% and 2.97%. Moisture values below 3% are beneficial for maintaining the bioactive properties of propolis [64].

The water activity of propolis is an important factor in determining its therapeutic potential. The a_w_ values do not provide a suitable environment for most microorganisms [65].

The ash content of propolis differs according to geographical area and plant type. Moroccan propolis has an ash content between 0.72% and 5.01% [66] and that from Argentina varies between 0.98% and 7.18% [67]. Our results fall between the values of 4.38% and 6.31%.

The content of phenols in propolis can vary widely depending on factors like geographical origin, plant sources, and the extraction method used. Generally, in European propolis, the values are between 150 and 300 mg GAE/g [68,69]. In Brazilian propolis (especially the green type), the values are between 200 and 500 mg GAE/g [70,71]. In other tropical regions, these values can vary between 50 and 600 mg GAE/g, depending on the local flora [72,73].

The total flavonoid content in European propolis is reported to range between 20 and 100 mg QE/g are reported [74,75], whereas Brazilian propolis, particularly green propolis, exhibits values between 30 and 300 mg QE/g [76,77]. For other tropical regions, the values registered were between 10 and 400 mg QE/g [78,79].

All tested honey and propolis samples demonstrated both antibacterial and antifungal activity, even if some of the honey samples did not act on all the strains examined. Even if some strains, especially *Salmonella* spp., were resistant to the activity of honey, the antibacterial and antifungal effect of all samples were demonstrated. Propolis samples exhibited greater antimicrobial activity compared to honey, with inhibition zone diameters often exceeding those of the control antibiotic or antifungal agents. This study once again confirmed the greater activity of propolis against Gram-positive than Gram-negative bacteria, which was already known [41].

The importance of this study lies in the fact that it has been demonstrated once again that honey and propolis from Romania have powerful antibacterial and antifungal effects. In the growing trend of utilizing antimicrobial compounds from natural sources, natural antimicrobial agents, especially those found in food with potential biomedical applications, are of significant interest.

### 3.1. Two-Way ANOVA Test

A statistical analysis was performed considering both the microbial strains and the geographical origin of bee products (honey, respectively propolis) to determine whether the diameter of inhibition zone (DIZ) is influenced by these two factors.

Analysis of variance (ANOVA) conducted to evaluate whether the correlation between the DIZ for the microbial strains and bee products was statistically significant at the 0.05 confidence level. The results are presented in Table 9.

Multiple comparisons were performed among the experimental data. Comparisons between honey and propolis samples and the diameter of the inhibition area for the studied strains, including bacterial and fungal strains, were conducted using a two-way analysis of variance (ANOVA). The analysis was performed on the two factors: bee products from Alba County, Romania, and the diameter of the inhibition area for the studied bacterial and fungal strains to find the effect of each factor, as well as any interactions between the independent variables.

In the case of bacterial strains, since F_col,H_ = 3.09 and F_col,P_ = 2.14 were both greater than F_0.05_ = 2.01, the null hypothesis that the mean values of the columns are equal was rejected. It was concluded that the honey and propolis samples from different areas of Alba County, Romania, distinctly influenced the diameter of the inhibition zone. At the same time, because F_row,H_ = 51.53 and F_row,P_ = 18.48 were also both greater than F_0.05_ = 2.58, the hypothesis that the mean values of the rows are equal was rejected, and it was concluded that the type of bacterial strain affected the diameter of inhibition areas. In the case of fungal strains, F_col,H_ = 0.46 and F_col,P_ = 1.22 were both smaller than F_0.05_ = 2.51. Thus, the null hypothesis that the mean values of the columns are equal was accepted. It was concluded that there are insufficient proofs to confirm that the propolis extracts influenced the diameters of the inhibition areas. Furthermore, since F_row,H_ = 2.41 and F_row,P_ = 1.80 were also smaller than F_0.05_ = 2.78, the hypothesis that the mean values of the rows are equal was also accepted, and it was concluded that only the type of strain did not affect the inhibition areas. The significance level was α = 0.05.

### 3.2. Pearson Correlation

The Pearson correlation analysis is presented in Table 10 and Table 11. The purpose was to evaluate the relationship between the content of flavonoids and phenol in the honey and propolis samples and the microbial strains (bacterial—Table 9 and fungal—Table 10) used.

In the case of honey, a high positive correlation was recorded between the flavonoid content and the DIZ for *S. aureus* and *S. epidermis* (Pearson correlation coefficient, r = 0.77, respectively 0.72). In addition, statistically significant correlations were found between the flavonoids and the DIZ for the following strains: *E. coli*, *S. Typhimurium*, *S. enteritidis*, and *P. fluorescens*. A very strong correlation was observed between the phenol content and the DIZ for the *E. coli* (r = 0.96) and *S. enteritidis* (r = 0.91) strains, followed *by P. aeruginosa*, *S. anatum*, and *S. Typhimurium*.

In the case of propolis samples, there was a positive correlation between the flavonoid content and the DIZ for *S. Typhimurium* (r = 0.71) and a moderate correlation in the case of *S. aureus* (r = 0.68). A statically significant correlation was noted between the phenols and the DIZ for following strains: *E. coli* (r = 0.95) and *S. Typhimurium* (r = 0.72).

The correlations between the inhibition zone diameter of the strains and the phenol content were more pronounced than those observed for the flavonoid content in both honey and propolis samples.

In the case of testing the activity of honey samples against fungal strains, there was a very high positive correlation between the flavonoids and the DIZ for *A. niger* and *F. oxysporum*, and a high correlation was noted in the case of *C. albicans* and *A. flavus*. In the case of phenolic compounds, the correlations were weaker, the most representative was noted for *A. alternata*. For propolis samples, the correlations between the DIZ and flavonoids were low and moderate, indicating, in general, a positive linear relationship between the two variables (Pearson correlation coefficient 0.68 for *A. niger*). The correlations between the DIZ and the phenols were similar. However, a very high correlation was noted in the case of *F. oxysporum* (Pearson correlation coefficient, r = 0.98), and high and moderate positive correlations were noted for other strains (*A. niger*, r = 0.69 and *A. flavus*, r = 0.58).

Overall, regardless of the microbial strains, the strength of the association is weaker for propolis samples compared to honey samples.

The findings of this study indicate that the chemical analysis of bee products from Alba County, Romania, reveals significant amounts of flavonoids and phenols. Along with their antimicrobial effects, honey and propolis can potentially be used as alternative medicines due to their therapeutic potential and may serve as a valuable source for generating functional foods.

## 4. Materials and Methods

The bee products were obtained from Romanian bee (*Apis mellifera carpatica*) living in specific pedoclimatic conditions (temperate continental climate) and melliferous plants from the Carpathian area. The samples were procured in 2023 directly from beekeepers without any prior heat treatment. The samples of unpasteurized polyfloral honey (H) and raw brown propolis (P) were obtained from the same apiary. The honey was collected in sterile glass jars after extraction. The floral source of honey was established by beekeepers. This was based on the existing flora, the location of the apiary, and the organoleptic qualities of the honey. The honey samples were stored at room temperature and in the dark until analysis. Propolis was harvested from the hive by scraping the wood with a stainless-steel spatula. The samples were stored at −20 °C until analysis.

**Figure 1:**
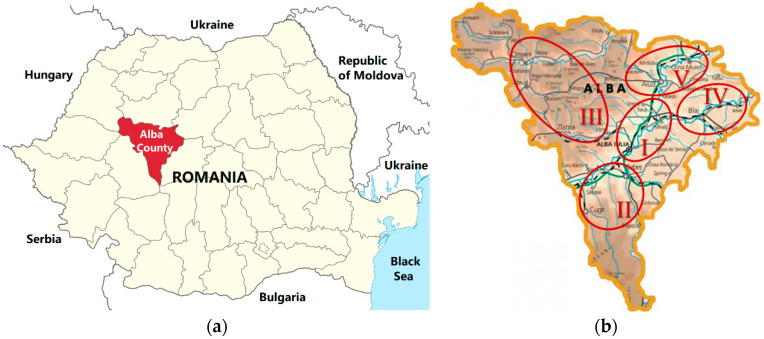
The map of Romania (**a**) and the distribution of the sampling areas of beekeeping products in the five areas of Alba County (**b**).

Figure 1 shows the map of Romania and Alba County, which was divided into five distinct sampling areas.

The investigated areas were established, taking into account the following elements of the apiary: the specific fauna and the flowering period, the climatic factors with the meteorological conditions (temperature, humidity, precipitation), the relief of the geographical area, the composition of the soil, and demographic considerations (Table 12).

Analysis of Honey and Propolis

The determination of the water content in honey was carried out with a Pocket Digital Refractometer PAL-22S (ATAGO, Tokyo, Japan). The moisture content of propolis was determined by measuring weight loss after heating in an oven at 105 °C ± 2 °C until a constant weight was achieved [51].

Water activity (A_w_) was measured using 10 g of sample (honey and propolis) with an Aquaspector apparatus AQS-2-TC (Nagy Messsysteme GmbH, Gäufelden, Germany) [80].

Determination of ash (total mineral substances). In a crucible, 10 g of sample (honey/propolis) was introduced, evaporated using a water bath, and carbonized in a low flame. This was followed by calcination at about 525 °C until a constant mass was obtained [81].

pH. Briefly, 10 g of honey was dissolved in 75 mL carbon dioxide-free water. The pH value was measured using a pH-meter inoLab^®^ pH 730 (Xylem Analytics, Weilheim, Germany) [82].

Determination of acidity. Here, 10 g of honey was dissolved in 50 mL of distilled water brought to 40–50 °C. The mix was titrated with 0.1N NaOH solution until the appearance of a pink color that persisted for 30 s in the presence of phenolphthalein, 1% alcoholic solution [81].

The HMF content was determined according to the White method. The absorbance of the honey samples was measured against the reference solution at 284 nm and 336 nm with a spectrophotometer (Lambda 20—Perkin Elmer UV/VIS, Washington, DC, USA). The HMF content was expressed in mg/kg honey [83].

To determine the total phenolic content (phenols) in samples (honey and propolis), the Folin–Ciocalteu method was used. The TPC was measured by interpolating the absorbance of the honey based on a calibration curve constructed with standard gallic acid. Total phenolic content was expressed as mg gallic acid equivalents per 100 g of honey (mg GAE/100 g of honey) [84] and mg gallic acid equivalents per 1 g of propolis (mg GAE/g of propolis) [85].

The total flavonoid content (flavonoids) in samples (honey and propolis) was measured using the aluminum chloride spectrophotometric assay. Total flavonoid content was expressed as mg of quercetin equivalents per 100 g of honey (mg QE/100 g H) [86] and quercetin equivalents per 1 g of propolis (mg EQ/g P) [87].

Preparation of Aqueous Propolis Extracts

The aqueous extracts of propolis were prepared following the method described in our previous study [88]. In summary, aqueous suspensions of propolis powder were refluxed for one hour, followed by two rounds of centrifugation. The mixture was then heated until 80% of the initial volume had evaporated, reaching the boiling point of water. The resulting aqueous extracts were stored in a dark, dry environment until needed. To test antifungal activity, samples were prepared at a concentration of 0.1 g/mL.

Microorganism Cultures

All strains used in this study were sourced from Thermo Fisher Scientific, Inc. (Waltham, MA, USA) and MicroBioLogics, Inc. (St. Cloud, MN, USA).

To assess the antibacterial activity of honey and propolis extracts, 12 bacterial strains were used, including 7 Gram-negative and 5 Gram-positive strains: *Escherichia coli* (ATCC 25922), *Salmonella typhimurium* (ATCC 14028), *Salmonella enteritidis* (ATCC 13076), *Salmonella anatum* (ATCC 9270), *Salmonella choleraesuis* (ATCC 7001), *Pseudomonas aeruginosa* (ATCC 27853), *Pseudomonas fluorescens* (ATCC 13525), *Staphylococcus aureus* (ATCC 25923), *Staphylococcus epidermidis* (ATCC 14990), *Bacillus cereus* (ATCC 11788), *Bacillus subtilis* (ATCC 6633), and *Listeria monocytogenes* (ATCC 19115). To obtain bacterial cultures, 3–5 colonies from each bacterial strain were dispersed in 10 mL of nutrient broth (Mikrobiologie Labor-Technik, Arad, Romania) and incubated for 18 ± 2 h at 37 ± 1 °C. The turbidity of the cell suspensions was measured using a McFarland Densitometer (Mettler Toledo, Columbus, OH, USA) and adjusted to a 0.5 McFarland standard, which corresponds approximately to a homogeneous suspension of 1.5 × 10^8^ CFU (colony forming units)/mL.

The antifungal activity of honey and propolis extracts was also tested using 7 fungal strains: *Candida albicans* (ATCC 10239), *Aspergillus niger* (derived from ATCC 16888), *Aspergillus flavus* (ATCC 9643), *Penicillium chrysogenum* (ATCC 10106), *Rhizopus stolonifer* (ATCC 14037), *Fusarium oxysporum* (ATCC 48112), and *Alternaria alternata* (TX 8025). For fungal cultures, colonies from each fungal strain were similarly dispersed in 10 mL of nutrient broth, incubated for 72 ± 2 h at 25 ± 1 °C, and adjusted to a 0.5 McFarland standard.

Determination of the Antimicrobial Properties of Honey and Propolis Extracts

The antimicrobial properties were evaluated using the disk diffusion method, following the procedures recommended by the CLSI [89]. The antimicrobial activity was assessed by measuring the diameters of the inhibition zones produced by various microbial strains. The diameter of these inhibition zones serves as a semi-quantitative indicator of the antimicrobial effectiveness.

Mueller–Hinton agar (Merck KGaA, Darmstadt, Germany) was used for culturing bacterial strains, while Sabouraud 4% dextrose agar (Merck KGaA) was used for fungal strains. The culture medium was poured into Petri dishes to a depth of approximately 4 mm (25 mL per plate). The surface of each Petri dish was inoculated by flooding with 1 mL of culture, which was then spread evenly across the surface. After inoculation, the plates were kept at 37 °C for 15 min to allow the inoculum to be absorbed into the agar.

For each honey sample, a sealed container of honey was liquefied in a water bath at 40–45 °C until all crystals were completely melted. Using a sterile stainless steel tube, 6.0 mm diameter circles were created by pressing and cutting into the culture medium of the Petri dishes. Each hole was then filled with 150 μL of undiluted honey. For each propolis sample, 50 μL of propolis extracts at a concentration of 0.1 g/mL (prepared as previously described) was added to ~6 mm filter paper discs. These discs were then sterilely placed on the surface of the culture medium and stored at 5 °C for 120 min. The holes and the discs were positioned at approximately equal distances from the edge of the plate and from each other.

The Petri dishes were incubated with their lids up for 18 ± 2 h at 37 ± 1 °C for bacterial growth and for 5 days at 25 ± 1 °C for fungal growth. Discs with 5 µg ciprofloxacin (Bio-Rad, Hercules, Marnes-la-Coquette, France) were used as a positive control for bacterial growth, and discs with 1 µg voriconazole (Bio-Rad, France) were used as positive controls for fungal growth. The antimicrobial activity was evaluated by measuring the diameter of the inhibition zones (in mm) using a DIN 862 ABS digital caliper (Fuzhou Conic Industrial Co., Ltd., Fuzhou, China).

Minimum Inhibitory Concentrations (MICs) of Honey and Propolis Extracts

The MIC values for the honey samples were determined using the micro-dilution broth method [90,91]. For honey samples, graduated doses of 10 g (*w*/*v*) of various types of honey were dissolved in sterile deionized water to create dilutions of 1/4, 1/8, 1/16, 1/32, and 1/64. For the propolis extracts, these were mixed with deionized water (*v*/*v*) to achieve final concentrations ranging from 50 mg/mL to 0.78 mg/mL for all propolis extracts.

Therefore, 10 μL of each diluted honey and diluted propolis extracts were added to the first line of the micro-titer wells containing 190 μL culture media, followed by 2-fold serial dilutions. Then, 100 μL volumes of the suspensions with fresh microbial cultures were inoculated into wells containing the honey or propolis extracts. The MIC results were recorded as the lowest concentration of honey/propolis extract that inhibits visible growth of a microorganism. A negative control (containing the tested extracts without the inoculum) was included on each microplate.

Minimum Bactericidal/Fungicidal Concentrations (MBCs/MFCs) of Honey and Propolis Extracts

The MBC/MFC values were determined by inoculating 10 µL of inoculum from the wells where the inhibitory effect was observed onto the Mueller-Hinton agar for bacterial growth and Sabouraud 4% dextrose agar plate for fungal growth. The plates prepared were incubated at 37 ± 1 °C for bacterial growth and for 5 days at 25 ± 1 °C for fungal growth. The MBCs/MFCs were determined for the lowest dilution at which microorganism growth was blocked.

## 5. Conclusions

This study allowed the comparative investigation of the antimicrobial activity of honey and propolis samples from different geographical areas of Alba County.

The quality parameters of the honey from Alba County, Romania, meet the requirements stipulated in the national and global standards, which ensure the authenticity and quality of the product.

The results demonstrate that honey and propolis samples from Alba County, Romania, exhibit antimicrobial activity against the tested strains. The two strains of *Staphylococcus* and *P. fluorescens* were the most sensitive to the effect of honey samples, while the strains of *L. monocytogenes*, *P. aeruginosa*, and *P. fluorescens* were particularly sensitive to propolis activity. Among the fungal strains, *F. oxysporum* exhibited the highest sensitivity to both honey and propolis.

The statistical analysis revealed a substantial correlation between the geographical origin of bee products and the bacterial strains, whereas no such correlation was proven for fungal strains.

## Figures and Tables

**Table 1:** Amount of honey produced both in Romania and Alba County [38,39].

Year	2013	2014	2015	2016	2017	2018	2019	2020	2021
Beehives in Romania, pcs	1354	1350	1392	1437	1602	1689	1843	1879	1903
Production in Romania, t	26,678	18,040	27,893	21,202	30,177	29,162	25,269	30,724	30,831
Production in Alba County, t	679	546	997	757	822	936	1105	1076	1090

**Table 2:** Physico-chemical parameters of honey (H) analyzed from Alba County.

Sample	Moisture Content (%)	Water Activity, a_w_	Ash Content (%)	pH	Acidity Meq/kg	HMF mg/100 g	Phenols (mg GAE/100 g)	Flavonoids (mg QE/100 g)
I H	15.23 ± 0.18	0.578 ± 0.013	0.20 ± 0.03	3.87 ± 0.34	20.8 ± 0.6	1.39 ± 0.06	82.14 ± 0.26	4.26 ± 0.18
II H	18.06 ± 0.35	0.582 ± 0.024	0.41 ± 0.06	4.02 ± 0.16	24.6 ± 0.9	1.13 ± 0.12	70.77 ± 0. 52	2.57 ± 0.05
III H	17.44 ± 0.42	0.577 ± 0.030	0.29 ± 0.02	4.14 ± 0.19	16.5 ± 0.7	0.47 ± 0.10	98.49 ± 1.03	5.35 ± 0.12
IV H	16.67 ± 0.20	0.591 ± 0.019	0.36 ± 0.07	3.98 ± 0.50	18.0 ± 0.2	1.21 ± 0.07	63.51 ± 0.28	3.29 ± 0.16
V H	14.51 ± 0.11	0.569 ± 0.027	0.45 ± 0.04	3.85 ± 0.22	14.3 ± 0.5	1.08 ± 0.09	64.20 ± 0.47	3.40 ± 0.31

Abbreviations: HMF—5-Hydroxymethylfurfural; GAE—gallic acid equivalents; QE—quercetin equivalents.

**Table 3:** Physico-chemical parameters of propolis samples (P) analyzed from Alba County.

Sample	Moisture Content (%)	Water Activity, a_w_	Ash Content (%)	Phenols (mg GAE/g)	Flavonoids (mg QE/g)
I P	4.38 ± 0.1	0.62 ± 0.20	3.21 ± 0.06	180.4 ± 4.28	76.22 ± 0.17
II P	5.07 ± 0.2	0.65 ± 0.14	2.48 ± 0.04	152.9 ± 3.71	71.53 ± 0.16
III P	6.31 ± 0.1	0.71 ± 0.12	3.02 ± 0.07	174.3 ± 5.02	80.72 ± 0.25
IV P	4.95 ± 0.1	0.66 ± 0.08	2.62 ± 0.08	149.2 ± 2.50	68.33 ± 0.14
V P	5.26 ± 0.1	0.70 ± 0.11	2.79 ± 0.05	165.6 ± 2.13	70.48 ± 0.30

Abbreviations: GAE—Gallic acid equivalents; QE—quercetin equivalents.

**Table 4:** Diameters of inhibition zones of bacterial strains (mm) obtained with polyfloral honey.

Microbial Strain	Sample					Ciprofloxacin 5 µg
	I H	II H	III H	IV H	V H	
*E. coli*	0	11	14	8	0	29
*S. typhimurium*	0	12	15	9	7	29
*S. enteritidis*	0	7	8	7	0	27
*S. anatum*	0	8	7	0	0	28
*S. choleraesuis*	0	10	10	0	7	28
*P. aeruginosa*	13	18	17	14	13	25
*P. fluorescens*	14	16	18	16	16	24
*S. aureus*	19	19	20	21	21	30
*S. epidermidis*	17	17	17	19	18	29
*B. cereus*	0	8	9	0	10	30
*B. subtilis*	17	18	19	11	12	30
*L. monocytogenes*	10	8	10	8	8	24

**Table 5:** Diameters of inhibition zones of bacterial strains (mm) obtained with propolis extracts.

Microbial Strain	Sample					Ciprofloxacin 5 µg
	I P	II P	III P	IV P	V P	
*E. coli*	18	27	32	22	18	29
*S. typhimurium*	18	26	30	25	24	29
*S. enteritidis*	15	19	25	17	15	27
*S. anatum*	17	26	22	16	19	28
*S. choleraesuis*	15	24	28	17	21	28
*P. aeruginosa*	28	32	29	27	27	25
*P. fluorescens*	27	33	33	28	29	24
*S. aureus*	22	27	26	30	27	30
*S. epidermidis*	26	24	20	31	29	29
*B. cereus*	24	26	27	24	29	30
*B. subtilis*	25	29	29	23	23	30
*L. monocytogenes*	31	29	30	28	28	24

**Table 6:** Diameters of inhibition zones of fungal strains (mm) obtained with polyfloral honey.

Fungal Strain	Sample					Voriconazole 1 µg
	I H	II H	III H	IV H	V H	
*C. albicans*	0	10	10	8	8	37
*A. niger*	8	9	10	10	9	45
*A. flavus*	9	9	10	10	10	43
*P. chrysogenum*	10	8	11	8	12	18
*R. stolonifer*	12	9	9	8	8	16
*F. oxysporum*	9	10	11	11	11	29
*A. alternata*	7	11	7	8	0	16

**Table 7:** Diameters of inhibition zones of fungal strains (mm) obtained with propolis extracts.

Fungal Strain	Sample					Voriconazole 1 µg
	I P	II P	III P	IV P	V P	
*C. albicans*	17	21	22	18	19	37
*A. niger*	15	23	26	25	21	45
*A. flavus*	17	25	27	24	23	43
*P. chrysogenum*	23	16	25	18	27	18
*R. stolonifer*	25	21	19	19	20	16
*F. oxysporum*	21	22	28	27	25	29
*A. alternata*	20	26	25	16	17	16

**Table 8:** Physical and chemical requirements for polyfloral honey sold on the Romanian market according to the 784/3-2009 national standard.

Parameter	Value			UM	Limit
	RO	EU	Codex		
Water	20	20	21	%	Maximum
Ash	0.5	0.5	0.6	%	Maximum
Sucrose	5	5	5	g/100 g	Maximum
Fructose and glucose content (sum of both)	70	60	65	g/100 g	Minimum
Free acid, ml NaOH sol 1N %	40	50	40	Meq/kg	Maximum
Water-insoluble content	0.1	0.1	0.1	g/100 g	Maximum
Electrical conductivity	-	0.8	-	mS/cm	Maximum
Diastase activity	10.9	8	8	Schade scale	Minimum
HMF	1.5 *	40	40	mg/100 g honey	Maximum

* Except for honey delivered in a jar, where the allowed value is 4 mg/100 g of honey; Abbreviations: RO—Romania—SR 784-2:2009 Honey Part 2: Quality requirements at sale [52]; EU—European Union—Council Directive 2001/110/EC relating to honey; Codex—Codex Alimentarius—Standard for Honey CXS 12-1981.

**Table 9:** Statistical results of the two-way analysis of variance (ANOVA): strains and the bee product.

Strains	Source of Variance	df	SS	MS	*F*-Ratio _calc._	F_0.05 prob._	Remark (prob > F)
Bacterial	Between samples, H	4	315.07	28.64	3.09	2.01	Significant
	Between samples, P		238.57	21.69	2.14	2.01	Significant
	Between strains, H	11	1911.78	477.95	51.53	2.58	Significant
	Between strains, H		750.53	187.63	18.48	2.58	Significant
	Errors, H	-	408.13	-	-	-	-
	Errors, P	-	446.63	-	-	-	-
	Total, H	44	9.28	-	-	-	-
	Total, P		10.15	-	-	-	-
Fungal	Between samples, H	4	16.86	2.81	0.46	2.51	Not significant
	Between samples, P		89.03	14.84	1.22	2.51	Not significant
	Between strains, H	6	59.49	14.87	2.41	2.78	Not significant
	Between strains, P		88.00	22.00	1.8	2.78	Not significant
	Errors, H	-	147.94	-	-	-	-
	Errors, P	-	292.57	-	-	-	-
	Total, H	24	6.16	-	-	-	-
	Total, P		12.19	-	-	-	-

Abbreviations: df—Degree of freedom, SS—sum of square, MS—mean square; *F*-ratio _calc._—calculated F statistic; F_0.05 prob_.—F-table of critical values for a significance level = 0.05.

**Table 10:** The Pearson correlation coefficients for the relationship between the flavonoid and phenol content in honey and propolis samples and the types of bacterial strains.

Bacterial Strains	Honey		Propolis	
	Flavonoids	Phenols	Flavonoids	Phenols
*E. coli*	0.637	0.966	0.460	0.957
*S. typhimurium*	0.695	0.775	0.713	0.728
*S. enteritidis*	0.613	0.911	0.184	0.550
*S. anatum*	0.055	0.817	0.250	−0.274
*S. choleraesuis*	0.136	0.464	0.051	0.362
*P. aeruginosa*	0.175	0.855	0.411	−0.477
*P. fluorescens*	0.667	0.672	0.277	0.133
*S. aureus*	0.776	−0.287	0.682	0.479
*S. epidermis*	0.721	−0.315	0.157	−0.068
*B. cereus*	0.167	0.158	−0.461	0.199
*B. subtilis*	−0.519	0.621	0.191	−0.095
*L. monocytogenes*	−0.539	0.287	−0.406	−0.267

**Table 11:** The Pearson correlation coefficients for the relationship between the flavonoid and phenol content in honey and propolis samples and the types of fungal strains.

Fungal Strains	Honey		Propolis	
	Flavonoids	Phenols	Flavonoids	Phenols
*C. albicans*	0.721	0.528	0.182	0.299
*A. niger*	0.950	0.591	0.568	0.699
*A. flavus*	0.789	0.026	0.449	0.582
*P. chrysogenum*	−0.185	−0.365	−0.917	0.353
*R. stolonifer*	−0.909	−0.106	−0.399	−0.757
*F. oxysporum*	0.912	0.194	0.142	0.981
*A. alternata*	−0.047	0.705	0.144	−0.201

**Table 12:** Geographical location of beekeeping products sampled in the five areas of Alba County.

Sample	Apiary Location Area	Geographical Origin	Latitude	Longitude	Landforms
I	Alba Iulia—Teiuș	Sântimbru	46°07′36″ N	23°37′38″ E	Plain
II	Sebeș—Cugir	Șibot	45°56′27″ N	23°20′10″ E	Sub-mountainous
III	Cîmpeni—Zlatna	Abrud	46°16′53″ N	23°03′39″ E	Mountainous
IV	Blaj	Valea Lungă	46°07′52″ N	24°04′48″ E	Hilly
V	Aiud—Ocna Mureș	Ciumbrud	46°18′29″ N	23°45′44″ E	Hilly

## Data Availability

Data are contained within the article and Supplementary Materials.

## Supplementary Materials

The following supporting information can be downloaded at: https://www.mdpi.com/article/10.3390/antibiotics13100952/s1. Table S1: Minimum Inhibitory Concentration (MIC) and Minimum Bactericidal Concentration (MBC) of Honey samples for bacterial strains. Table S2: Minimum Inhibitory Concentration (MIC) and Minimum Fungicidal Concentration (MBC) of Honey samples for fungal strains. Table S3: Minimum Inhibitory Concentration (MIC) and Minimum Bactericidal Concentration (MBC) of Propolis Extracts for bacterial strains. Table S4: Minimum Inhibitory Concentration (MIC) and Minimum Fungicidal Concentration (MFC) of Propolis Extracts for fungal strains.
