# Supplementary material for: Antimicrobial Activity of Honey and Propolis from Alba County, Romania
Source: Antibiotics (Basel). 2024 Oct 10;13(10):952. doi: 10.3390/antibiotics13100952 (PMC11504579; doi:10.3390/antibiotics13100952)
Supplement: Supplementary file 1 [file antibiotics-13-00952-s001.zip › antibiotics-3238873-supplementary.pdf]

Contents

Minimum Inhibitory Concentration (MIC) and Minimum Bactericidal/Fungicidal Concentration (MBC/MFC) of Honey samples: **Supplementary Tables S1-S2**
Minimum Inhibitory Concentration (MIC) and Minimum Bactericidal/Fungicidal Concentration (MBC/MFC) of Propolis Extracts: **Supplementary Tables S3-S4**

Supplementary Table S1. Minimum Inhibitory Concentration (MIC) and Minimum Bactericidal Concentration (MBC) of Honey samples for bacterial strains

**Microbial Strain**	**MIC/MBC (%w/v)**									
	I H		II H		III H		IV H		V H	
	MIC	MBC	MIC	MBC	MIC	MBC	MIC	MBC	MIC	MBC
E. coli	-	-	12.5	25	12.5	25	12.5	25	-	25
S. typhimurium	-	-	12.5	25	12.5	25	12.5	25	25	25
S. enteritidis	-	-	25	50	12.5	50	25	50	-	-
S. anatum	-	-	12.5	25	12.5	50	-	-	-	-
S. choleraesuis	-	-	12.5	25	12.5	25	-	-	12.5	25
P. aeruginosa	25	50	6.25	12.5	12.5	25	12.5	25	12.5	25
P. fluorescens	12.5	25	12.5	25	12.5	12.5	12.5	12.5	12.5	25
S. aureus	6.25	12.5	6.25	12.5	6.25	12.5	12.5	25	6.25	12.5
S. epidermidis	12.5	25	12.5	25	12.5	25	6.25	25	12.5	25
B. cereus	-	-	25	25	25	25	-	-	25	25
B. subtilis	12.5	25	12.5	25	12.5	50	25	25	25	50
L. monocytogenes	12.5	25	6.25	25	12.5	25	12.5	25	12.5	25

Supplementary Table S2. Minimum Inhibitory Concentration (MIC) and Minimum Fungicidal Concentration (MBC) of Honey samples for fungal strains

**Microbial Strain**	**MIC/MFC (%w/v)**									
	I H		II H		III H		IV H		V H	
	MIC	MFC	MIC	MFC	MIC	MFC	MIC	MFC	MIC	MFC
C. albicans	-	-	12.5	25	12.5	12.5	12.5	50	12.5	50
A. niger	12.5	25	12.5	12.5	6.25	12.5	12.5	25	12.5	25
A. flavus	12.5	25	12.5	25	12.5	12.5	12.5	25	12.5	12.5
P. chrysogenum	12.5	12.5	12.5	25	3.12	12.5	12.5	25	6.25	12.5
R. stolonifer	6.25	12.5	12.5	12.5	12.5	12.5	12.5	25	12.5	25
F. oxysporum	12.5	12.5	3.12	12.5	6.25	12.5	6.25	25	12.5	25
A. alternata	25	50	6.25	12.5	25	50	12.5	25	-	-

Supplementary Table S3. Minimum Inhibitory Concentration (MIC) and Minimum Bactericidal Concentration (MBC) of Propolis Extracts for bacterial strains

**Microbial Strain**	**MIC/MBC (mg/mL)**									
	I P		II P		III P		IV P		V P	
	MIC	MBC	MIC	MBC	MIC	MBC	MIC	MBC	MIC	MBC
E. coli	12.5	25	3.12	12.5	6.25	12.5	12.5	12.5	25	50
S. typhimurium	12.5	12.5	6.25	12.5	3.12	12.5	12.5	12.5	3.12	6.25
S. enteritidis	25	50	12.5	25	6.25	12.5	25	25	12.5	25
S. anatum	12.5	12.5	6.25	12.5	3.12	25	25	25	12.5	25
S. choleraesuis	25	25	6.25	12.5	6.25	12.5	12.5	12.5	6.25	12.5
P. aeruginosa	12.5	12.5	1.56	6.25	1.56	6.25	12.5	25	6.25	25
P. fluorescens	12.5	25	1.56	12.5	6.25	12.5	12.5	25	1.56	6.25
S. aureus	12.5	25	6.25	6,25	6.25	12.5	12.5	25	3.12	6.25
S. epidermidis	12.5	25	6.25	6.25	12.5	12.5	12.5	12.5	3.12	12.5
B. cereus	12.5	25	6.25	12.5	6.25	12.5	12.5	25	1.56	6.25
B. subtilis	12.5	12.5	3.12	6.25	1.56	6.25	12.5	25	6.25	25
L. monocytogenes	12.5	12.5	3.12	12.5	1.56	6.25	12.5	25	3.12	12.5

Supplementary Table S4. Minimum Inhibitory Concentration (MIC) and Minimum Fungicidal Concentration (MFC) of Propolis Extracts for fungal strains

**Microbial Strain**	**MIC/MFC (mg/mL)**									
	I P		II P		III P		IV P		V P	
	MIC	MFC	MIC	MFC	MIC	MFC	MIC	MFC	MIC	MFC
C. albicans	12.5	12.5	6.25	6.25	6.25	12.5	25	25	12.5	25
A. niger	12.5	25	6.25	6.25	3.12	6.25	12.5	25	6.25	12.5
A. flavus	25	25	3.12	6.25	3.12	6.25	12.5	12.5	3.12	12.5
P. chrysogenum	12.5	12.5	6.25	12.5	6.25	6.25	12.5	25	3.12	12.5
R. stolonifer	12.5	12.5	6.25	6.25	6.25	12.5	12.5	25	6.25	6.25
F. oxysporum	12.5	25	6.25	12.5	1.56	6.25	12.5	12.5	3.12	12.5
A. alternata	12.5	25	3.12	12.5	3.12	6.25	12.5	25	6.25	12.5
